# Fine tuning chloroplast movements through physical interactions between phototropins

**DOI:** 10.1093/jxb/erw265

**Published:** 2016-07-12

**Authors:** Olga Sztatelman, Justyna Łabuz, Paweł Hermanowicz, Agnieszka Katarzyna Banaś, Aneta Bażant, Piotr Zgłobicki, Chhavi Aggarwal, Marcin Nadzieja, Weronika Krzeszowiec, Wojciech Strzałka, Halina Gabryś

**Affiliations:** ^1^Department of Plant Biotechnology, Faculty of Biochemistry, Biophysics and Biotechnology, Jagiellonian University, Gronostajowa 7, 30-387 Krakow, Poland; ^2^Institute of Biochemistry and Biophysics, Polish Academy of Sciences, Pawińskiego 5a, 02-106 Warsaw, Poland; ^3^Malopolska Centre of Biotechnology, Jagiellonian University, Gronostajowa 7A, 30-387 Krakow, Poland.

**Keywords:** *Arabidopsis thaliana*, blue light, chloroplast movements, light pulses, phototropin1, phototropin2, protein phosphatase 2A.

## Abstract

Phototropins are plant photoreceptors which regulate numerous responses to blue light, including chloroplast relocation. Weak blue light induces chloroplast accumulation, whereas strong light leads to an avoidance response. Two Arabidopsis phototropins are characterized by different light sensitivities. Under continuous light, both can elicit chloroplast accumulation, but the avoidance response is controlled solely by phot2. As well as continuous light, brief light pulses also induce chloroplast displacements. Pulses of 0.1s and 0.2s of fluence rate saturating the avoidance response lead to transient chloroplast accumulation. Longer pulses (up to 20s) trigger a biphasic response, namely transient avoidance followed by transient accumulation. This work presents a detailed study of transient chloroplast responses in Arabidopsis. Phototropin mutants display altered chloroplast movements as compared with the wild type: *phot1* is characterized by weaker responses, while *phot2* exhibits enhanced chloroplast accumulation, especially after 0.1s and 0.2s pulses. To determine the cause of these differences, the abundance and phosphorylation levels of both phototropins, as well as the interactions between phototropin molecules are examined. The formation of phototropin homo- and heterocomplexes is the most plausible explanation of the observed phenomena. The physiological consequences of this interplay are discussed, suggesting the universal character of this mechanism that fine-tunes plant reactions to blue light. Additionally, responses in mutants of different protein phosphatase 2A subunits are examined to assess the role of protein phosphorylation in signaling of chloroplast movements.

## Highlight

Physical interactions between phototropin molecules can alter their signaling outcomes, providing the means for the plant to fine-tune its blue light responses.

## Introduction

Light is a crucial factor in plant life. Apart from supplying energy for photosynthesis, it also provides information about the environment. To detect the quality and quantity of incident light, multiple specialized proteins have evolved in plants. Phototropins (phots) are blue light/UV-A photoreceptors which primarily control several key responses important for the optimization of light capture. These include phototropism, leaf expansion and positioning, the light-driven opening of stomata, and chloroplast relocation (Christie, 2007).

The Arabidopsis genome encodes two phototropins, PHOT1 and PHOT2. Both consist of an N-terminal photosensory part and a C-terminal protein Ser/Thr kinase domain. The photosensory part is made up of two LOV (light oxygen voltage-regulated) domains, which non-covalently bind FMN chromophores (reviewed in Christie, 2007). In darkness, the LOV2 domain acts as a kinase inhibitor (Matsuoka and Tokutomi, 2005). Upon light absorption, a covalent bond is formed between the FMN chromophore and a conserved cysteine within the LOV domain. This leads to conformational changes resulting in kinase activation (Tokutomi *et al.*, 2008). The first substrate of the kinase is the phototropin itself, since autophosphorylation is the initial step of signaling (Inoue *et al.*, 2008). Serine residues in the activation loop of the phot1 kinase domain are indispensable for signal transduction (Inoue *et al.*, 2008). Their homologs seem to be important for phot2 signaling (Inoue *et al.*, 2011). Most of the identified phot1 and phot2 phosphorylation sites lie in the N-terminus or in the hinge region between LOV domains (Salomon *et al.*, 2003; Sullivan *et al.*, 2008; Inoue *et al.*, 2011). They do not appear to be essential for photoreceptor-mediated responses, but rather modulate interactions with other proteins, as in the case of 14-3-3 proteins (Inoue *et al.*, 2008). Apart from autophosphorylation, transphosphorylation between phot1 molecules (Kaiserli *et al.*, 2009) as well as between phot1 and phot2 (Cho *et al.*, 2007) has been reported. Dephosphorylation of the conserved serine residues in the phot1 kinase blocks the signal transduction from this photoreceptor (Inoue *et al.*, 2008). Phot1-specific phosphatases remain unknown. The only identified protein phosphatase responsible for phot2 dephosphorylation is protein phosphatase 2A (PP2A). This is a trimeric enzyme, consisting of a catalytic subunit C, a scaffolding subunit A, and a regulatory subunit B. Each subunit type is encoded by multiple genes in Arabidopsis: five catalytic, three scaffolding, and 17 regulatory subunits, which can be further divided into B, B', and B'' subunit families. The resulting trimeric holoenzyme is highly variable, which provides the molecular basis for its specific functions via the modification of different targets (Trotta *et al.*, 2011). Regulatory subunits determine the substrate specificity of the holoenzyme (Uhrig *et al.*, 2013). The scaffolding subunit A1 was originally identified as regulating auxin transport in the roots and named ROOTS CURL IN NAPHTHYLPHTHALAMIC ACID1 (RCN1) (Garbers *et al.*, 1996). This subunit interacts specifically with phot2 and leads to its dephosphorylation. As a consequence of enhanced phot2 phosphorylation, the *rcn1* mutant exhibits enhanced phototropism and stomatal movements in the *phot1* background (Tseng and Briggs, 2010).

Both phototropins are bound to the plasma membrane in darkness (Sakamoto and Briggs, 2002; Kong *et al.*, 2006). After blue irradiation, a fraction of phot1 is released into the cytoplasm (Sakamoto and Briggs, 2002), whereas phot2 associates with the Golgi apparatus (Kong *et al.*, 2006; Aggarwal *et al.*, 2014). The change in localization of both phototropins requires the C-terminal part of the protein. On the other hand, the dimerization of phot1 (Kaiserli *et al.*, 2009) is probably determined by the N-terminal part of the photoreceptor, as isolated LOV domains tend to form dimers (Salomon *et al.*, 2004; Katsura *et al.*, 2009; Nakasone *et al.*, 2014).

Chloroplast movements are among the responses mediated by phototropins. In many plant species, chloroplast positioning is regulated by the intensity of incident light (Zurzycki, 1980). In Arabidopsis under weak blue light (0.08–4 μmol m^−2^ s^−1^), chloroplasts gather along the cell walls perpendicular to the light direction in order to maximize light capture. This is called the accumulation response. When blue light becomes stronger (>20 μmol m^−2^ s^−1^), chloroplasts migrate to the cell walls parallel to the incident light, which is known as the avoidance response (Trojan and Gabryś, 1996; Jarillo *et al.*, 2001; Sakai *et al.*, 2001). Intermediate blue light fluence rates (5–10 μmol m^−2^ s^−1^) trigger a biphasic response, initial chloroplast avoidance being followed by the accumulation reaction (Luesse *et al.*, 2010).

Chloroplast positioning in Arabidopsis depends on phot1 and phot2. Both photoreceptors mediate the accumulation response, but only phot2 is able to elicit chloroplast avoidance (Jarillo *et al.*, 2001; Kagawa *et al.*, 2001; Sakai *et al.*, 2001). Arabidopsis phototropin mutants are characterized by the altered sensitivity of chloroplasts to blue light. A *phot2* mutant in which only phot1 is active shows chloroplast accumulation regardless of blue light intensity starting from 0.08 μmol m^−2^ s^−1^. At high fluence rates of blue light (40–100 μmol m^−2^ s^−1^), a small biphasic response is generated, which is interpreted as the result of a residual avoidance response just after the onset of light (Luesse *et al.*, 2010). In the *phot1* mutant, which bears only phot2, both responses occur, although accumulation is triggered at higher blue light intensities (2–20 μmol m^−2^ s^−1^) than in the wild type (Sakai *et al.*, 2001). No directional chloroplast movements are observed in the double phototropin mutant (Sakai *et al.*, 2001).

Chloroplast relocations are confined to and depend on the local light conditions inside the cell. Partial irradiation of the cell with strong blue light (120 μmol m^−2^ s^−1^) causes simultaneous avoidance and accumulation responses of chloroplasts in the same cell (Kagawa and Wada, 2000). Chloroplasts which are directly exposed to strong light move away from the light spot. Chloroplasts outside the strong blue light beam accumulate at its border but do not enter into the illuminated part of the cell.

Chloroplast movements are not only induced by continuous light. Brief pulses of light followed by darkness lead to transient rearrangements of chloroplasts (Gabryś *et al.*, 1981). In *Tradescantia albiflora* and *Lemna trisulca*, short pulses (20ms to 1s) of strong blue light (120 μmol m^−2^ s^−1^) induce transient chloroplast accumulation. Pulses of longer duration (3–100s) result in a biphasic response of chloroplasts, initial transient avoidance being followed by accumulation. The responses to pulses obey the reciprocity law; that is, the same energy fluence brings about a response of the same amplitude and kinetics irrespective of the pulse duration and fluence rate (Gabryś *et al.*, 1981).

In the current study, chloroplast relocation in response to light pulses is examined in the Arabidopsis wild type, and phototropin and PP2A subunit mutants. The expression of phototropins as well as their dephosphorylation are analyzed in mutants exhibiting differences in chloroplast relocation as compared with the wild type. Moreover, phototropin molecules are shown to form homo- and heterocomplexes *in planta.* The results provide evidence that phototropins co-operate rather than compete in eliciting chloroplast movements.

## Materials and methods

### Plant material and cultivation conditions

All mutants used in this study were T-DNA-containing SALK lines in the Col-0 background that have been described before: *phot1* (At3g45780), SALK_088841 (Lehmann *et al.*, 2011); *phot2* (At5g58140), *npl1-1* (Jarillo *et al.*, 2001); *rcn1-6* (At1g25490), SALK_059903 (Blakeslee *et al.*, 2007); *pp2a-b'*γ (At4g15415), SALK_039172 (Trotta *et al.*, 2011); *pp2a-b'ζ1-1* (At3g21650), SALK_107944C (Rasool *et al.*, 2014); and *pp2a-2* (At1g10430), SALK_150673 (Wen *et al.*, 2012). The *rcn1-6* allele was selected instead of the *rcn1-1* allele used by Tseng and Briggs (2010) because of its genetic background. RCN1 protein has not been detected in extracts of *rcn1-6* seedlings (Blakeslee *et al.*, 2007). *phot1*, *phot2*, *pp2a-b'*γ, *pp2a-b'ζ1-1*, and *pp2a-b’*γ/*pp2a-b’ζ1-1* were acquired from the respective authors. SALK_059903C and SALK_150673 lines were purchased from the Nottingham Arabidopsis Stock Centre, and their homozygosity was confirmed/identified by PCR analysis according to the standard protocol (Alonso *et al.*, 2003) using the primers listed in Supplementary Table S1 at *JXB* online.

Seeds were sown in Jiffy-7 pots (Jiffy Products International AS) and placed at 4 °C for 2 d. Plants were grown in a growth chamber (Sanyo MLR 350H) at 23 °C, 80% relative humidity, with a photoperiod of 10h light and 14h darkness, at 70 μmol m^−2^ s^−1^ light supplied by Sanyo MLR 350H lamps. Four- to five-week-old plants were used for the experiments.

### Photometric measurements of chloroplast movements

Chloroplast movements were quantified using a photometric method (Walczak and Gabryś, 1980), which is based on recording the changes in weak red light transmittance (0.3 µmol m^−2^ s^−1^, 660nm, modulated at a frequency of 800 Hz), which are caused by chloroplast relocation. Chloroplast movements were induced by 120 µmol m^−2^ s^−1^ blue light (LED Luxeon Royal Blue LXHL-FR5C, Philips Lumiled Lighting Comp, 460nm). Plants were dark-adapted for 16h before the measurement. A detached leaf was mounted in a holder and the initial transmittance level was recorded for 5min. Then a pulse of blue light was applied, followed by the recording of changes in transmittance for another 40min or 120min. After measuring the response to the shortest pulse, the leaf was kept in darkness to regain the initial (dark) position of chloroplasts. Meanwhile another (typically control) leaf was assessed. Subsequently, the former leaf was used for measuring responses to longer pulses. Ideally, a whole series of six pulses of different duration (0.1, 0.2, 1, 2, 10, and 20s) were applied to a single leaf during 1 d.

For quantification of chloroplast movements in response to continuous blue light, plants were dark-adapted for 16h and detached leaves were used. The dark transmittance level was recorded for 20min and leaves were exposed to weak blue light (1.6 µmol m^−2^ s^−1^) for 45min, followed by strong blue light (120 µmol m^−2^ s^−1^) for 45min.

Photometric curves were analyzed using a custom-written Mathematica (Wolfram Research, USA) package. Responses to pulses and continuous illumination were characterized by their amplitudes and rates. Amplitudes of transmittance changes were calculated relative to the dark transmittance level. The maximal rate of transmittance change was calculated as the derivative of the photometric curve, using a Savitzky–Golay filter, with the window width set to 3min. To better characterize the dynamics of responses to pulses, the times between the pulse onset and the maximum (transient avoidance) or minimum (transient accumulation) of transmittance were calculated. In the accumulation phase of the responses to 20s pulses, the transmittance often reached a plateau and no distinct minimum was noticeable. In such cases, the time between the pulse onset and the beginning of the plateau was calculated.

The statistical significance of the effects of plant line and light conditions was assessed with one- or two-way (as specified in the text) ANOVA, followed by Dunnett’s test, used for pairwise comparisons between wild-type plants, treated as a control, and mutant plants. The *P*-values reported in the text and figures are adjusted for multiple comparison. All statistical calculations were performed using the R software.

### Determination of protein and mRNA levels

Arabidopsis wild-type plants and *phot1*, *phot2*, and *rcn1-6* mutants were dark-adapted overnight. To determine the protein and mRNA content in leaves, plants were irradiated with white light of 120 µmol m^−2^ s^−1^ (Fytoscope FS130 Photon System Instruments) for 3h. Illuminated and control, dark-adapted leaves were collected at the same time and immediately frozen in liquid nitrogen. For the dephosphorylation experiments, whole plants were illuminated with blue light of 120 µmol m^−2^ s^−1^ (LXHL-PR09, Ledium Ltd) for 1h. A dark-adapted control and a sample from time 0, just after illumination, were collected. The remaining illuminated plants were transferred to darkness and samples were taken after 20, 40, 60, 90, and 120min. All samples were frozen in liquid nitrogen immediately after collection.

RNA isolation and real-time PCR were performed as described elsewhere (Łabuz *et al.*, 2012). Briefly, RNA isolated with a Spectrum Plant Total Kit (Sigma-Aldrich) was reverse transcribed with a RevertAid M-MuLV Reverse Transcriptase Kit (Thermo Scientific) using random hexamer primers. SYBR Green JumpStart Taq ReadyMix (Sigma-Aldrich) and a thermal cycler (Rotor-Gene 6000, Corbett Research) were used to perform the real-time PCR analysis. Primer sequences for *PHOT1* and *PHOT2* are listed in Łabuz *et al.* (2012); for reference genes, *UBC* and *PDF2* are listed in Czechowski *et al.* (2005). The relative expression of each gene in a sample was determined using the mean value of *Ct* for all samples as a reference. Normalization of phototropin expression levels was performed using normalization factors calculated by geNorm v3.4 (Vandesompele *et al.*, 2002). For each combination of light conditions (light/darkness) and plant line (wild type/*rcn1*/*phot1*/*phot2*), two independent samples (biological replicates) were prepared; each sample contained leaves pooled from four different plants. Transcript levels were measured in three technical replicates for each sample.

To determine the mRNA level of *PP2A-2* in wild-type and homozygous *pp2a-2* (SALK_150673) leaves, RNA was extracted and reverse-transcribed as described above. PCR was performed using gene-specific primers given by Wen *et al.* (2012). 18S RNA served as an internal standard with a 3:7 primer:competimer ratio (QuantumRNA™ 18S RNA, Ambion). PCR conditions were as follows: 3min at 98 °C and 33 cycles of 15s at 95 °C, 15s at 55 °C, and 60s at 72 °C.

For protein determination, Arabidopsis leaves were homogenized, weighed, and adjusted to an equal mass. Proteins were extracted according to the protocol of (Sakamoto and Briggs, 2002). SDS–PAGE was performed on 7.5% polyacrylamide gels with subsequent semi-dry protein transfer (Bio-Rad). A duplicate polyacrylamide gel was stained with a Coomassie Brilliant Blue (CBB) solution to check the quantity of proteins in each sample. After the transfer, the membrane was blocked with 5% milk in phosphate-buffered saline (PBS), 0.05% Tween-20, and incubated with primary antibodies in the same solution at 4 °C overnight. Anti-PHOT1 (AS10 720) and anti-PHOT2 (AS10 721) antibodies described in Łabuz *et al.* (2015) were obtained from Agrisera. Anti-PHOT2 antibodies were used at a dilution of 1:5000, and anti-PHOT1 antibodies at 1:300 (a purified fraction). After washing, the membranes were incubated with secondary antibodies [goat anti-rabbit horseradish peroxidase (HRP)-conjugated IgG, Agrisera] at a dilution of 1:25 000. The signal was detected with a Clarity Western ECL Blotting Substrate (Bio-Rad) using the BioSpectrum Imaging System (UVP Ultra-Violet Products Ltd). Intensities of the chemiluminescent signal were compared with the total protein amounts in given samples visualized by CBB staining of the gel.

### Determination of the phototropin phosphorylation level

Proteins were extracted from leaves in the following buffer: 0.1M Tris–HCl, 3% SDS, 2mM phenylmethylsulfonyl fluoride (PMSF) for 3min in 80 °C and centrifuged at 16 000 *g*, 4 °C for 10min (3-30KS, Sigma). A 100 μl aliquot of the supernatant was ultrafiltrated twice with water (W4502, Sigma) using Amicon Ultra-0.5 Centrifugal Filter 30K devices (Millipore) according to the manufacturer’s instructions. The protein concentration was estimated using the Bradford method (Bradford, 1976). A 10 μg aliquot of total protein was dephosphorylated using 12.5U of Fast AP alkaline phosphatase (Thermo Scientific) at 37 °C for 1h. SDS–PAGE was performed in a Laemmli system (Laemmli, 1970) on 7.5% polyacrylamide gels containing 50 μmol l^–1^ Phos-tag (SuperSep Phos-tag, Wako). The gels were incubated twice in transfer buffer with 10mM EDTA for 10min followed by 10min in transfer buffer before semi-dry protein transfer (Bio-Rad). Phototropin detection was performed as described above. To assess the protein amounts, membranes were stripped with Restore Plus Western Blot Stripping Buffer (Thermo Scientific) and probed with anti-actin antibody (AS132640, Agrisera) diluted 1:2000 in 5% milk PBS-T at room temperature for 1h, followed by secondary antibody incubation and ECL detection.

### Bimolecular fluorescence complementation (BiFC)

Constructs for BiFC analysis were prepared using vectors described by Karimi *et al.* (2007) and the MultiSite Gateway cloning system (Invitrogen). The PUNI51 plasmids U09177 and U24125 were used as templates to amplify the coding sequences of *PHOT1* and *PHOT2*, respectively. Both plasmids were obtained from the Arabidopsis Biological Resource Center (ABRC). All constructs were cloned with the Easy-A High Fidelity polymerase (Stratagene) and their identities were verified by sequencing. The transient transformation of *Nicotiana benthamiana* leaves was performed as described in Aggarwal *et al.* (2014). For the negative BiFC control, plasmids encoding the N- or C-terminal green fluorescent protein (GFP) fragment fused to the first 150 amino acids from the N-terminal part of the red fluorescent protein (RFP) protein were used (Strzalka *et al.*, 2015). The primers and plasmids used for cloning are listed in Supplemetnary Tables S2 and S3. Microscopy was performed with an LSM 880 laser scanning microscope (Carl Zeiss, Jena, Germany). A Plan-Neofluar ×40, 1.3 NA objective was used with oil immersion. An argon laser line of 488nm was used for excitation. Emission within the range of 493–597nm was recorded as the green channel, and emission in the range of 638–721nm as the red channel.

The expression of proteins in the BiFC assay was determined using the western blot protocol described above. After the transfer and blocking, the membranes were incubated overnight in 5% milk in PBS-T with the antibodies. To detect the N-terminal part of GFP, Living Colors GFP Monoclonal Antibody (Clonetech, catalog no. 632375) was used at a dilution of 1:10 000. The C-terminal part of GFP was recognized by Santa Cruz Biotechnology GFP mouse monoclonal antibody (B-2) (catalog no. sc-9996) at a dilution of 1:200.

### Split-ubiquitin-based membrane yeast two-hybrid (MYTH) system

Protein interactions were tested using the split-ubiquitin-based MYTH system (MoBiTec), with introduced Gateway cloning sequences (Strzalka *et al.*, 2015). Bait (pDHB1Gateway) and prey (pPR3-NGateway) vectors containing full-length phototropins or their N- or C-terminal domains (according to Aihara *et al.*, 2008) were prepared as described for BiFC vectors, using the primers given in Supplementary Table S2. Yeast transformation and handling were described elsewhere (Strzalka *et al.*, 2015). For scoring interactions, transformed yeast plated on agar plates were kept in 30 °C either in darkness or under blue light (~20 μmol m^−2^ s^−1^, 470nm) for 3 d. Each experiment was repeated at least three times.

## Results

### Chloroplast movements in response to light pulses in wild-type *Arabidopsis thaliana*

**Fig. 1.**
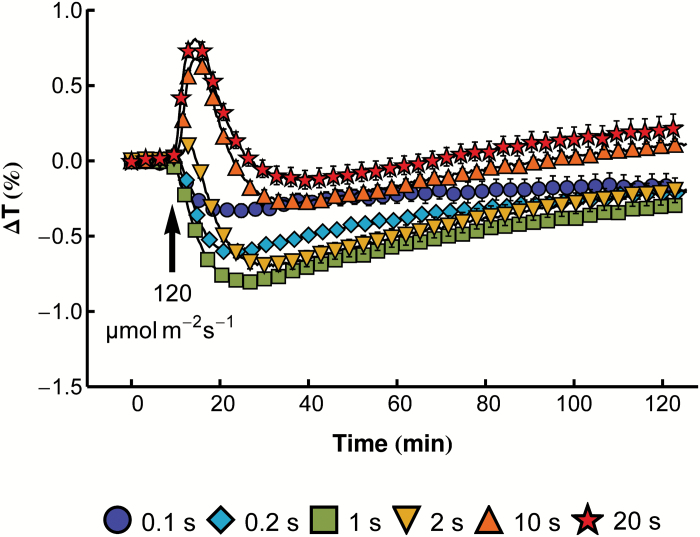
Chloroplast movements in response to strong blue light pulses in wild-type Arabidopsis. Time course of changes in red light transmittance were recorded before and after a blue light pulse of 120 µmol m^−2^ s^−1^ and duration specified in the figure. Each data point is an average of at least 16 measurements. Error bars show the SE.

Chloroplast relocation after light pulses provides insights into the signaling mechanism of these movements, but to date a detailed analysis is lacking for *A. thaliana*. Blue light pulses of 120 µmol m^−2^ s^−1^ were chosen to study chloroplast responses in Arabidopsis leaves, as this intensity saturates chloroplast avoidance when applied as continuous light. In wild-type leaves, very short pulses of 0.1, 0.2, and 1s elicited transient accumulation responses (Fig. 1). The 1s light pulse produced the largest amplitude of chloroplast accumulation. Longer pulses (2, 10, and 20s) resulted in a biphasic response of chloroplasts, with initial transient avoidance followed by transient accumulation. The accumulation amplitude was smaller than that observed after the pulse of 1s. After the 20s pulse, chloroplasts returned to the dark position within the period of observation (120min). The recording time of 40min was used in further studies because it covers the most characteristic part of the response.

### Chloroplast responses to light pulses in phototropin mutants

**Fig. 2.**
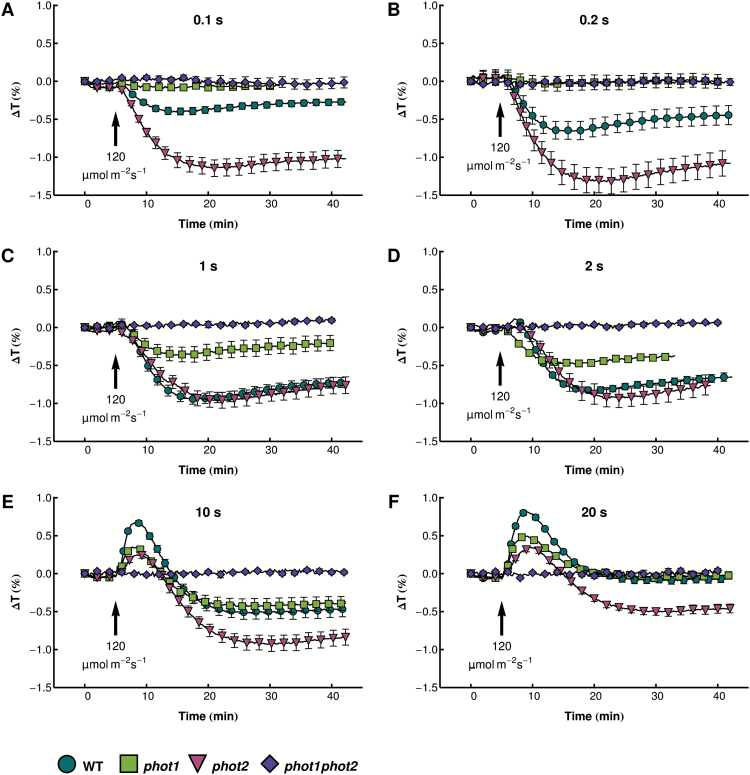
Chloroplast movements in response to strong blue light pulses in wild-type Arabidopsis and phototropin mutants. Time course of changes in red light transmittance were recorded before and after a blue light pulse of 120 µmol m^−2^ s^−1^ and duration specified in the figure. Each data point is an average of at least eight measurements. Error bars show the SE.

**Fig. 3.**
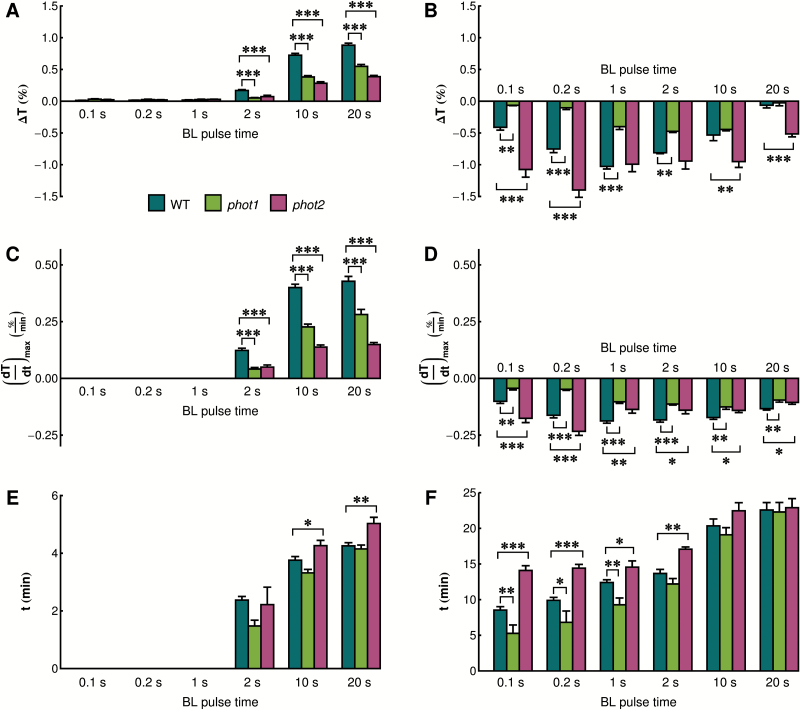
Parameters of chloroplast movements after strong blue light pulses in wild-type Arabidopsis and phototropin mutants. The parameters were calculated for the avoidance (A, C, E) and accumulation (B, D, F) parts of the curves. (A and B) Maximal amplitude of the responses, (C and D) maximal velocity of the responses, (E and F) time needed to reach the maximum of the response. Each data point is an average of at least eight measurements. Error bars show the SE. Asterisks indicate statistically significant differences: *P=0.01–0.05; **P=0.001–0.01, ***P<0.001.

To understand the differences in the light sensitivities of phototropin mutants with regard to chloroplast movements, the responses to short blue light pulses were analyzed in *phot1*, *phot2*, and *phot1phot2* mutant plants (Fig. 2). The *phot1phot2* double mutant did not show any movements triggered by blue light pulses, proving that the observed chloroplast relocation relies solely on phototropins. Similarly, the responses of the *phot1* mutant (in which only phot2 is active) to the shortest pulses (0.1 and 0.2s) were barely above the noise level. Longer pulses (1s and 2s) triggered weak transient chloroplast accumulation. After 10s and 20s pulses, biphasic responses were observed, with amplitudes lower than in the wild type for the avoidance phase and comparable with the wild type for the accumulation phase. ANOVA revealed that the presence of phototropin mutations and pulse duration significantly affected the transient chloroplast responses, both in their accumulation (ANOVA for amplitude: effect of plant line *F*_2,234_=108.48, *P*<0.0001, effect of pulse duration *F*_5,234_=32.11, *P*<0.0001) and the avoidance phase (ANOVA for amplitude: effect of plant line *F*_2,125_=146.58, *P*<0.0001, effect of pulse duration *F*_2,125_=283.48, *P*<0.0001). The amplitudes of transmission changes for both phases are shown in Fig 3A and B. The differences between *phot1* and the wild type were statistically significant for all responses, except for accumulation after the longest (10s and 20s) pulses. The velocity of transmission changes (Fig. 3C, D) was slower in the *phot1* mutant than in the wild type for all pulses tested. Times needed to reach maximal avoidance were similar for wild-type and *phot1* plants (Fig. 3E) for all light pulses tested. Times needed to reach maximal accumulation were significantly shorter for the *phot1* mutant for pulses not longer than 1s (Fig. 3F).

In contrast, the *phot2* mutant (with only phot1 active) showed enhanced accumulation responses after the shortest (0.1s and 0.2s) and longest (10s and 20s) pulses (Figs 2, 3A, B). Despite the lack of phot2, this mutant underwent a transient avoidance response after longer pulses. This response was significantly weaker than that observed in the wild type, but comparable with that in the *phot1* mutant. The accumulation response was significantly faster for the shortest pulses (0.1s and 0.2s), but significantly slower for the longer ones (Fig. 3C). The *phot2* mutant was also characterized by the extended times needed to reach the maximal responses for both chloroplast accumulation after shorter pulses and avoidance after longer pulses (Fig. 3E, F).

### Chloroplast responses to light pulses in mutants of different PP2A subunits

**Fig. 4.**
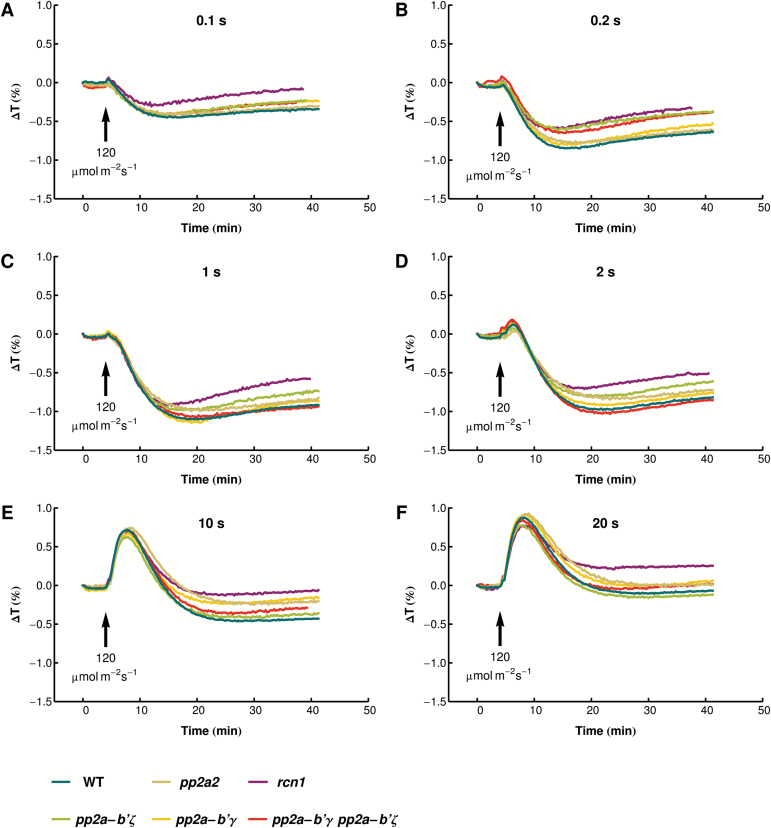
Chloroplast movements in response to strong blue light pulses in wild-type Arabidopsis and mutants in selected subunits of PP2A phosphatase. Time course of changes in red light transmittance were recorded before and after a blue light pulse of 120 µmol m^−2^ s^−1^ and the duration specified in the figure. Each data point is an average of at least seven measurements. The figure is line-only for clarity; a version with error bars is included as Supplementary Fig. S1.

**Fig. 5.**
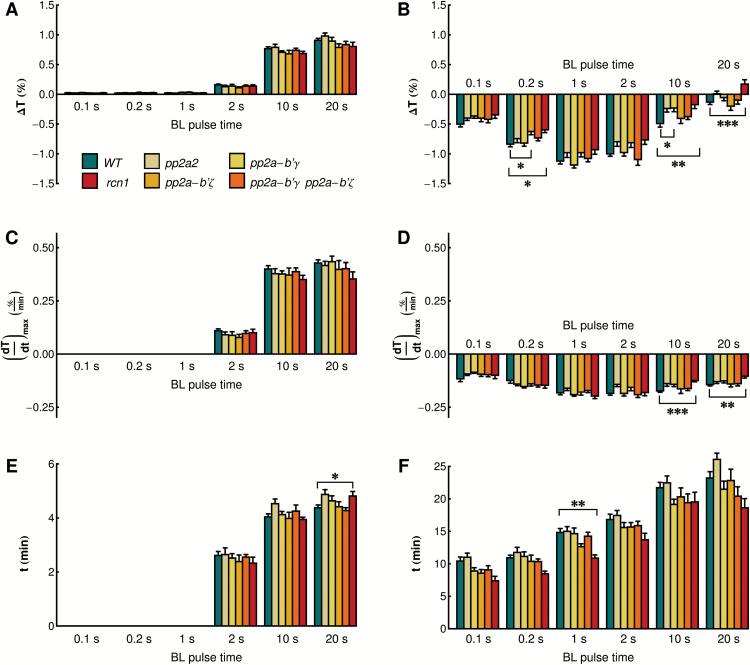
Parameters of chloroplast movements after strong blue light pulses in wild-type Arabidopsis and mutants in selected subunits of PP2A phosphatase. The parameters were calculated for the avoidance (A, C, E) and accumulation (B, D, F) parts of the curves. (A, B) Maximal amplitude of the reaction, (C, D) maximal velocity of the reaction, (E, F) time needed to reach the maximum of the response. Each data point is an average of at least seven measurements. Error bars show the SE. Asterisks indicate statistically significant differences: *P=0.01–0.05; **P=0.001–0.01, ***P<0.001

To link phototropin signaling leading to chloroplast movements with phototropin phosphorylation status, responses to light pulses were examined in mutants of different PP2A subunits, *rcn1* (the scaffolding subunit A1 shown to interact with phot2) and regulatory B' subunits, γ and ζ, which are involved in high light tolerance (Konert *et al.*, 2015). ANOVA revealed that the chloroplast responses were significantly affected by pulse duration and the presence of the *rcn1* mutation, in both the accumulation (ANOVA for amplitude: effect of plant line *F*_5,455_=15.46, *P*<0.0001, effect of pulse duration *F*_5,455_=201.74, *P*<0.0001) and the avoidance phase (ANOVA for amplitude: effect of plant line *F*_5,248_=7.20, *P*<0.0001, effect of pulse duration *F*_2,248_=492.46, *P*<0.0001). Chloroplast relocation in mutants of the B' subunits was comparable with that in the wild type (Figs 4, 5; for clarity Fig. 4 is line-only, a version with error bars is presented in Supplementary Fig. S1). The post-hoc Dunnett’s test showed that significance of the effect of plant line seen in ANOVA was due to the *rcn1* mutant, which showed a lower amplitude and a decrease in the kinetics of the accumulation response after the longest pulses (10s and 20s) as compared with the wild type. The time needed to reach the maximal accumulation was generally shorter in this mutant than in the wild type, although this difference was not statistically significant for most pulses. A slight elongation of the time needed to reach maximal avoidance for the longest pulse was also observed, the *rcn1* mutant thus showing a shift in the balance between chloroplast accumulation and avoidance towards the latter, mimicking the effect of a longer light pulse. Recently, a mutant of the PP2A catalytic subunit *pp2a-2* has been shown to have weaker chloroplast movements in response to strong continuous light (Wen *et al.*, 2012). Surprisingly, in our hands, the same *pp2a-2* mutant—the homozygous SALK_150673 line (Supplementary Fig. S2A)—displayed responses to blue light pulses comparable with wild-type plants (Figs 4, 5). Chloroplast relocation under continuous light was indistinguishable from that in the wild type (Supplementary Fig. S2B). The lack of difference between the wild type and the *pp2a-2* mutant might result from leaky expression of *PP2A-2* (Supplementary Fig. S2C).

### Phototropin expression in mutants with altered chloroplast responses to blue light pulses

**Fig. 6.**
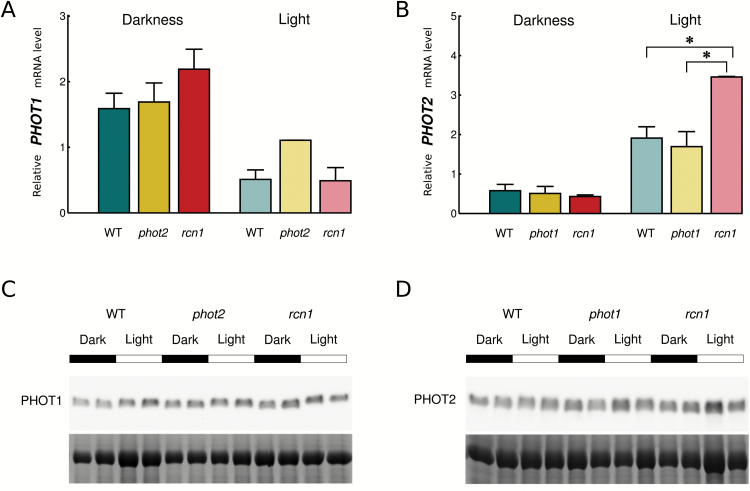
Profiles of phototropin1 (A) and phototropin2 (B) expression in darkened and light-exposed (120 µmol m^−2^ s^−1^ and 3h) Arabidopsis wild-type and mutant (*phot1*, *phot2*, and *rcn1*) leaves at the mRNA level. Each point represents the average obtained from at least nine leaves of different plants. Error bars show the SE. Asterisks indicate statistically significant differences between samples *P=0.01–0.05. (C and D) A representative western blot showing the expression of PHOT1 (C) and PHOT2 (D) in wild-type and mutant plants. Proteins stained with CBB are shown as the well loading reference.

To investigate whether altered chloroplast relocation in the face of blue light pulses was due to differences in phototropin expression, both mRNA and protein levels were examined in the leaves of the wild type and selected mutants with altered chloroplast movements, namely *phot1*, *phot2*, and *rcn1* (Fig. 6). Both phototropin proteins accumulated to a higher level in the *rcn1* mutant, irrespective of light conditions. These differences were not a simple result of changes in the transcript level. In wild-type plants the expression of *PHOT2* was up-regulated by light, while the expression of *PHOT1* was down-regulated. The mRNA level of *PHOT2* after light treatment was higher in the *rcn1* mutant than in the wild type, in contrast to the *phot1* mutant where no statistically significant differences were observed. The amount of *PHOT1* mRNA in *rcn1* after light treatment was comparable with that in wild-type plants. The level of the *PHOT1* transcript in the *phot2* mutant was influenced by light to a lesser extent than in the wild type. At the protein level, the *phot2* mutant had more phot1 after light exposure. In the *phot1* mutant, the amount of phot2 was comparable with that in the wild type. The differences, although observable, were not substantial.

### Phototropin dephosphorylation in mutants with altered responses to blue light pulses

**Fig. 7.**
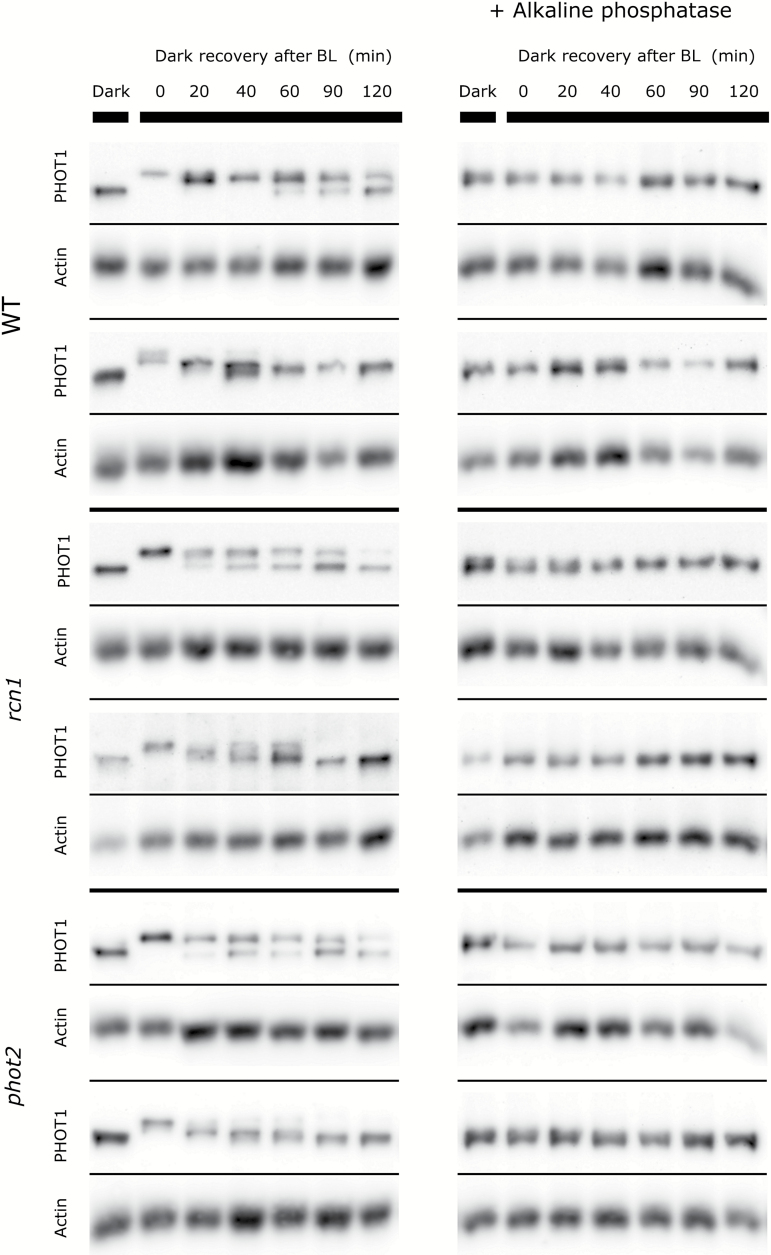
Representative dephosphorylation profiles of phototropin1 after blue light exposure (120 µmol m^−2^ s^−1^ and 1h) in Arabidopsis wild-type and mutant (*phot2* and *rcn1*) leaves. Dark, a dark-adapted control; 0, a sample collected just after illumination. The duration of the incubation in the darkness after the end of the illumination is indicated in minutes. Phosphorylation leads to the shift of the phototropin band towards higher mass. Samples treated with alkaline phosphatase are shown on the right. Anti-actin blots are presented as the loading reference. The results represent two out of 4–5 independent biological replicates.

**Fig. 8.**
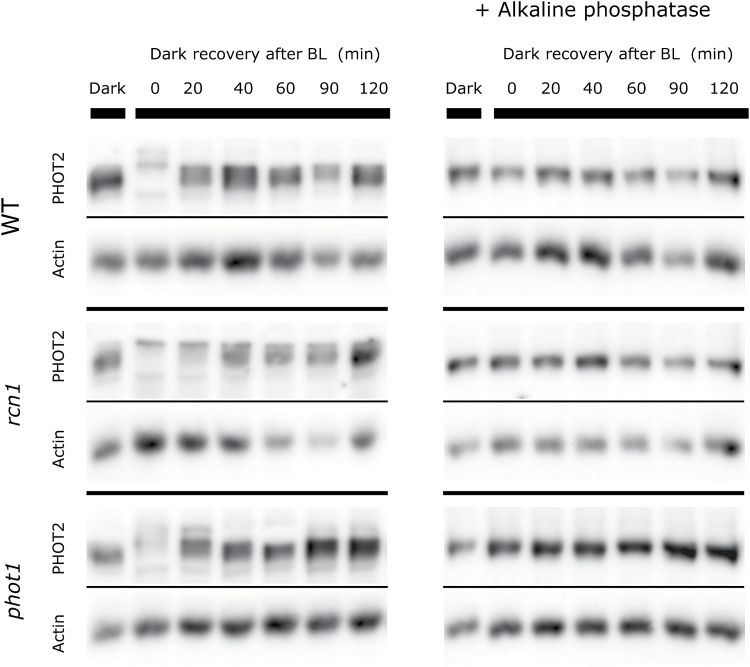
Representative dephosphorylation profiles of phototropin2 after blue light exposure (120 µmol m^−2^ s^−1^ and 1h) in Arabidopsis wild-type and mutant (*phot1* and *rcn1*) leaves. For further description, see the legend of Fig. 7. The results represent one of 3–4 independent biological replicates.

To assess the dephosphorylation dynamics of phototropins in the mutants (*phot1*, *phot2*, and *rcn1*), the decline of phosphorylation after saturating light treatment was estimated. Arabidopsis plants were first exposed to blue light of 120 µmol m^−2^ s^−1^ for 1h and then left in darkness for the specified period of time (up to 120min). The mobility shifts of phototropin bands after electrophoresis in the presence of Phos-tag were analyzed (Figs 7, 8). The shifts resulted from changes in phototropin phosphorylation, as they disappeared when samples were treated with alkaline phosphatase (Figs 7, 8). Two patterns of phot1 phosphorylation decay were observed: either a disappearance of the higher (phosphorylated) band and a reappearance of the lower (dephosphorylated) band or a gradual change in the mobility of the main band. No major differences between the wild type, and *phot2* and *rcn1* mutant lines were detected (Fig. 7). phot2 formed a wide band just after light treatment, which gave a weaker signal in blots as compared with the samples kept in darkness (Fig. 8). The density profiles of bands had several local maxima, indicating that phot2 exists in a variety of phosphorylated states in strong light. Similarly to phot1, clear reappearance of the lower (dephosphorylated) phot2 band was observed when leaves were transferred to darkness. No differences were observed between examined lines, except for the time point of 20min after switching off the light, when phot2 remained more phosphorylated in *phot1* and *rcn1* mutants as compared with the wild type. In general, phot1 phosphorylation persisted longer than that of phot2 in wild-type plants.

### Interactions between phototropin molecules

**Fig. 9.**
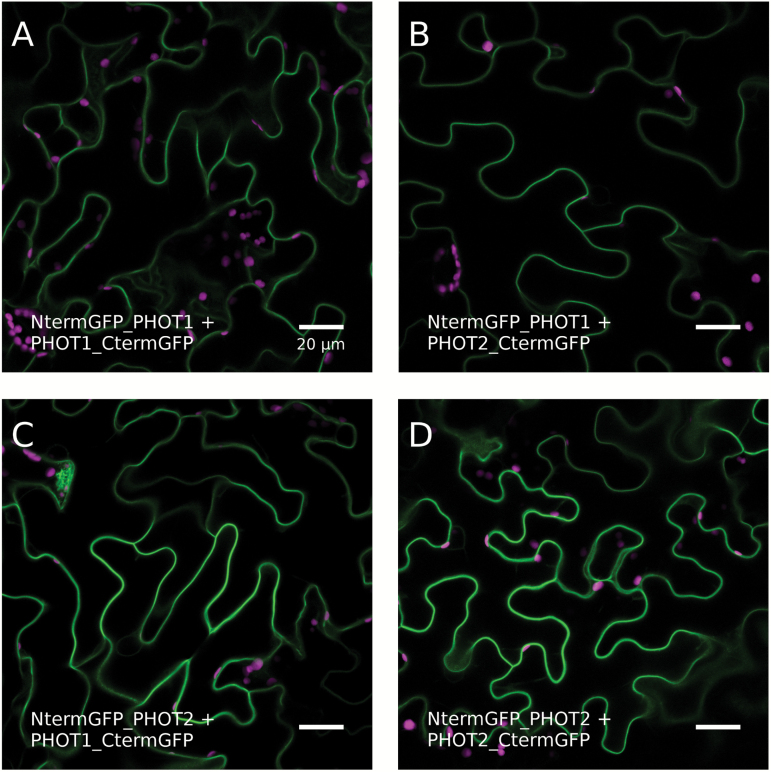
Confocal images of *N. benthamiana* epidermal cells transiently co-expressing phototropins fused with C(N)-terminal GFP fragments in the following configurations: NtermGFP_PHOT1 and PHOT1_CtermGFP (A), NtermGFP_PHOT1 and PHOT2_CtermGFP (B), NtermGFP_PHOT2 and PHOT1_CtermGFP (C), and NtermGFP_PHOT2 and PHOT2_CtermGFP (D). Chlorophyll autofluorescence is in magenta, and reconstituted GFP fluorescence is in green. Scale bar=20 µm. The results represent one of three independent biological replicates.

The differences in the magnitude of chloroplast accumulation after light pulses demonstrated for phototropin mutants can be plausibly explained if these proteins interact with each other. Hence, BiFC analysis was employed to examine the possibility of homo- and heterocomplex formation between phot1 and phot2 molecules. The formation of all types of these complexes was assessed upon transient expression in *N. benthamiana* epidermal cells. Green fluorescence was observed for PHOT1–PHOT1, PHOT2–PHOT2, and PHOT1–PHOT2 combinations in the following configurations: NtermGFP_PHOT1 and PHOT1_CtermGFP (Fig. 9A), NtermGFP_PHOT2 and PHOT1_CtermGFP (Fig. 9B), NtermGFP_PHOT1 and PHOT2_CtermGFP (Fig. 9C), and NtermGFP_PHOT2 and PHOT2_CtermGFP (Fig. 9D). Both phototropin homodimers, as well as heterodimers were localized in the proximity of the plasma membrane. The specificity of GFP reconstitution was tested using co-expression of the phototropin–N(C) GFP fragment with its N(C)terminal GFP counterpart fused with the first 150 amino acids of the RFP. None of such control pairs showed green fluorescence (Supplementary Fig. S3), indicating the specificity of phototropin dimer formation. The presence of recombinant proteins in transformed leaves was confirmed using anti-GFP antibodies (Supplementary Fig. S4).

**Fig. 10.**
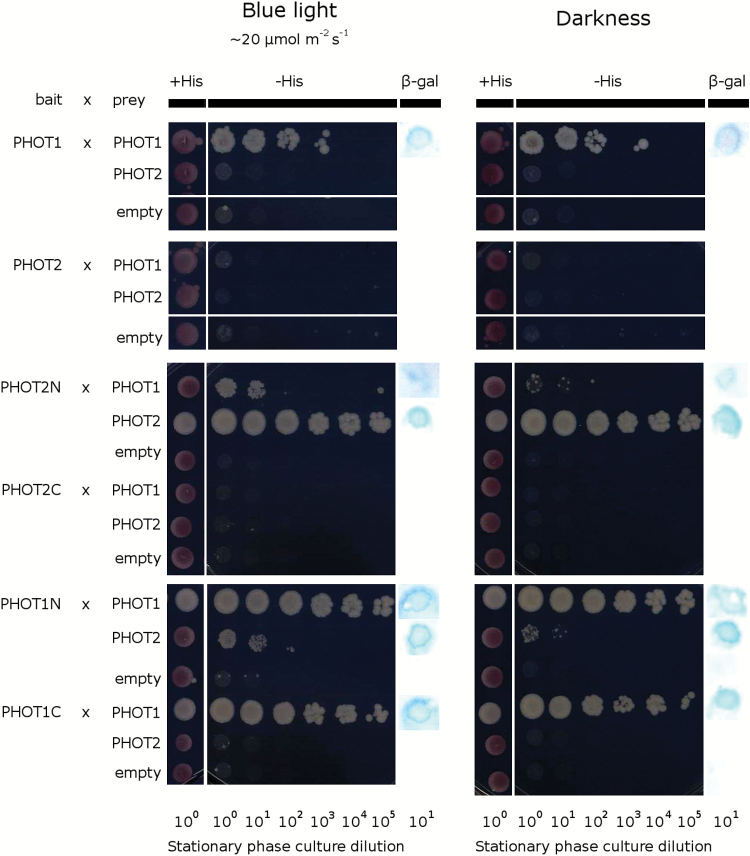
Phototropin interactions tested with MYTH assay. Full-length phototropins and their N/C-terminal parts were used as baits, and full-length phototropins only were used as preys. Overnight cultures of transformed yeasts were plated on the solid SC-Leu-Trp (+His) medium serving as a control, SC-Leu-Trp-His (-His) solid selection medium supplemented with 5mM 3-aminotriazole (3-AT), or YPAD solid medium to perform β-galactosidase filter lift-off assay. In each case, the yeast plated on solid media were cultured either in darkness or under blue light (~20 μmol m^−2^ s^−1^, 470nm) in 30 °C for 3 d. For all bait/prey constructs, a co-transformation with empty prey/bait vectors was performed to avoid false-positive signals being a result of a non-specific self-activation. The results represent one of at least three independent biological replicates.

In an alternative approach, a MYTH assay was performed to examine the interactions between phototropin molecules. When full-length phototropins were used as both prey and bait, the complex formation was observed only between two phot1 molecules (Fig. 10). As the results from BiFC and the MYTH assay were inconsistent, truncated versions of PHOTs were used as baits to test interactions with full-length proteins. When the N-terminal part of either PHOT1 (amino acids 1–619) or PHOT2 (amino acids 1–572) were used as baits, the interactions with both full-length photoreceptors were observed (Fig. 10). When C-terminal parts of the phototropins (PHOT1, amino acids 620–996; PHOT2, amino acids 573–915) were used as baits, only the interaction between PHOT1C and PHOT1 was observed. No complex formation was observed when full-length phototropins were used as baits for truncated phototropin preys (Supplementary Fig. S5). The interactions between phototropins were mostly independent of blue light. Only the heterodimer formation between PHOT1N and PHOT2 and between PHOT2N and PHOT1 was slightly stronger in the presence of blue light. These results suggest that in both homo- and heterocomplexes, phototropins interact mainly via their N-terminal part. However, phot1 molecules may also interact via the C-terminal part. Bait proteins in the MYTH system used in this study are membrane bound at the N-terminus and fused to a transcription factor at the C-terminus. This system allows examination of phototropin interactions near the plasma membrane. However, steric hindrance may lead to false negatives.

## Discussion

### Responses to light pulses as a tool for the analysis of signal transduction in chloroplast movements

The chloroplast accumulation response can be triggered with very short light pulses, while illumination with longer pulses results in a biphasic response—transient avoidance followed by an accumulation phase. The transient avoidance is faster, but more short-lived than accumulation. The high sensitivity of these responses to light makes the pulse-based method an excellent tool for studying the phototropin signaling mechanism.

Chloroplast responses to light pulses in Arabidopsis are similar to those observed for other plant species, reflecting their universal character (Gabryś *et al.*, 1981). It was proposed that the chloroplast position inside the cell depends on the level of an active state produced by a photoreceptor with a half-lifetime of the order of minutes (Gabryś *et al.*, 1981). Higher levels of this signaling state are needed for chloroplast avoidance; lower levels lead to accumulation. A level of signaling state sufficient to induce avoidance is produced by a strong light pulse that is long enough. The half-lifetime of this state was estimated to be 3min (Zurzycki *et al.*, 1983). Upon dark relaxation, the level of the signaling state drops and accumulation is induced. After the discovery and characterization of the photoreceptors responsible for chloroplast movements, this active state may be interpreted as activated phototropin itself. phot1 was shown to retain its autophosphorylation activity for several minutes after a light pulse (Kaiserli *et al.*, 2009). phot2 is characterized by a faster dark relaxation than phot1 (Christie *et al.*, 2002), so its signaling state is probably shorter lived. These properties of phototropins are in line with chloroplast responses to the shortest pulses. The accumulation response reaches its maximum earlier in the *phot1* mutant than in the *phot2* mutant (Fig. 3).

Microscopic observations of chloroplast relocations after switching off the strong light microbeam resemble the biphasic responses after longer pulses (Higa and Wada, 2015). Chloroplasts stay outside the previously irradiated area of the cell for a short time (3–4min). Then they move into that area for 19–28min. Those results were interpreted as the effect of both avoidance and accumulation signals being produced and competing under strong light, with the latter being longer lived but weaker. The signal lifetimes estimated by Higa and Wada (2015) are in good agreement with the times of maximal avoidance/accumulation after brief light pulses reported in this work. Similarly, biphasic responses to light pulses might result from the prevalence of the stronger avoidance signal over the weaker accumulation signal.

In wild-type and *phot1* plants, the accumulation phase of the response after a 10s or 20s pulse is much weaker than after shorter pulses. After a 20s pulse, the dark positioning is often restored without any transient accumulation. Thus, longer pulses must produce a signal suppressing chloroplast accumulation. Lack of suppression in *phot2* suggests that phot2 actively inhibits chloroplast accumulation after longer pulses. The LOV1 domain of the phot1 molecule has been shown to inhibit chloroplast accumulation under higher light intensities (Kaiserli *et al.*, 2009). The interplay of phototropins operating in one cell may be the second level of this accumulation control.

### Chloroplast responses to light pulses in phototropin mutants point to phototropin co-operation in chloroplast movement signaling

As both phototropins can elicit chloroplast accumulation, it may seem counterintuitive that after short pulses the *phot2* mutant exhibits stronger accumulation than the wild type. However, this result is consistent with chloroplast movements observed under low continuous light. *phot1* shows weaker accumulation, whereas in the *phot2* mutant this response is stronger than in the wild type under non-saturating light conditions (Luesse *et al.*, 2010). The effect has been attributed to the existence of two distinct and partially antagonistic signaling pathways originating from each phototropin. In this context, the balance between those signals determines the magnitude of chloroplast relocations.

The differences between the wild type and phototropin mutants in the accumulation reaction after the shortest light pulses might result from changes in phototropin levels, since photoreceptor abundance appears to regulate both the velocities and amplitudes of chloroplast movements (see discussion in Łabuz *et al.*, 2015). If the absence of one phototropin led to changes in the level of the other one, that would affect the phenotype. However, the expression of phot1 in the *phot2* mutant and phot2 in the *phot1* mutant is similar to that observed in the wild type (Fig. 6). The slight increase in the amount of phot1 after prolonged light treatment observed in the *phot2* mutant cannot account for the reactions to light pulses measured in dark-adapted plants.

The mutant phenotypes may also be explained as the consequences of phototropin interactions. Results of the MYTH assay indicate that truncated phototropins can interact with full-length phot1 and phot2 (Fig. 10). Whereas LOV dimer formation has been reported before (Nakasako *et al.*, 2004; Salomon *et al.*, 2004; Katsura *et al.*, 2009), the results presented here suggest that LOV domain dimerization can take place in the presence of full-length photoreceptor intramolecular interactions. Homo- and heterodimers of both phototropins are also observed *in planta* (Fig. 9). The submembrane localization of phot1/phot2 homodimers and phot1–phot2 heterodimers is the same as shown for single phototropin molecules. In wild-type plants, three types of phototropin complexes may form: homodimers of each phototropin (phot1–phot1 and phot2–phot2) and heterodimers (phot1–phot2). It can be hypothesized that following the absorption of light quanta a photoreceptor molecule transactivates its partner to amplify the signal. In weak light (or after a very brief pulse) phot1 is more likely to become activated due to its higher light sensitivity than phot2 (Christie *et al.*, 2002). The kinase activity of phot1 is stronger than that of phot2 (Aihara *et al.*, 2008). Thus, phot1 produces a very strong signal in homodimers, while that generated by heterodimers is weaker. Phot2 homodimers elicit the relatively weakest signal. As a result, in wild-type plants, the final outcome is a sum of signals from different types of phototropin complexes. In the *phot1* mutant, only phot2 homodimers exist, and these elicit only a relatively weak response (small amplitudes of the responses to the shortest light pulses, Fig. 2). In the *phot2* mutant, phot1 homodimers produce a very strong signal, not diluted by phot2-containing heterodimers. As a consequence, the *phot2* mutant exhibits a stronger accumulation response after short light pulses than the wild type (Fig. 2). Heterodimer formation may also explain the magnitude of chloroplast biphasic responses after the longest light pulses (10s and 20s). By forming heterodimers with phot2, phot1 strengthens the signal leading to chloroplast avoidance. Indeed, a higher amplitude of transient avoidance in response to light pulses is observed in wild-type plants as compared with the *phot1* mutant (Fig. 3A). In continuous light, this avoidance enhancement effect is observed at non-saturating light intensities (Luesse *et al.*, 2010; Łabuz *et al.*, 2015). These results suggest that phot1 fine-tunes the onset of chloroplast avoidance.

The postulated mechanism seems to be supported by previous studies. Individual LOV domains form dimers (Nakasako *et al.*, 2004; Salomon *et al.*, 2004; Katsura *et al.*, 2009). Dimerization and transphosphorylation between distinct phot1 molecules *in planta* have been shown by Kaiserli *et al.* (2009). Transphosphorylation of phot1 by phot2 has been demonstrated by Cho *et al.* (2007). Further, these authors observed a higher bending angle of seedlings bearing LOV-inactivated phot1 than those bearing LOV-inactivated phot2 in the double mutant background in some light intensities. The activity of LOV-inactivated photoreceptors was postulated to result from the cross-activation of mutated photoreceptors by leaky phot2. The enhanced reaction to light suggests that independently of its photosensing properties, phot1 has a higher activity level than phot2. Similar conclusions emerge from an examination of phenotypes elicited by chimeric phototropins, proteins consisting of the N-terminal part of phot1 fused with the C-terminal part of phot2, or vice versa. The results reported by Aihara *et al.* (2008) indicate that phot1 is more active independently of light sensitivity. Although the highest differences in light sensitivity originate from the N-terminal parts of chimeric photoreceptors, consistent with their photochemical properties, the C-terminal parts also enhance this sensitivity. The increased activity can prolong the lifetime of the signal leading to chloroplast movements, observed as longer times of transient accumulation after the shortest light pulses in the *phot2* mutant.

The hypothesis of phototropin co-operation provides a plausible interpretation of the physiological relevance of differences in the expression patterns of these photoreceptors. phot2 expression is mainly driven by light. This protein is practically absent in wild-type etiolated seedlings (Inoue *et al.*, 2011; Łabuz *et al.*, 2012), mimicking the situation in *phot2* mutant leaves. Phototropism in etiolated seedlings is the most sensitive phototropin-elicited reaction (Sakai *et al.*, 2001). The signal amplification is driven by phot1 alone and, owing to the lack of phot2, even the weakest light can be perceived. Light induces the production of phot2 and, in consequence, the sensitivity of the phototropin system decreases. Seedlings emerging from the soil need to sense the lowest light intensity to grow towards it. However, the light fluence rate sufficient for phototropism is way too low to support growth at later stages of development. Phototropins mediate reactions aimed at optimizing photosynthetic light capture (such as chloroplast accumulation); hence, to be cost-efficient they must operate under fluence rates which are effective for photosynthesis.

### The residual avoidance triggered by phot1

Although phot1 cannot elicit typical chloroplast avoidance in response to strong light (Sakai *et al.*, 2001), the *phot2* mutant displays a transient increase in leaf transmittance interpreted as a residual avoidance response (Luesse *et al.*, 2010; Łabuz *et al.*, 2015). Similarly, a biphasic response occurs in the *phot2* mutant after longer light pulses (10s and 20s), with transient avoidance followed by transient accumulation (Fig. 2). The amplitude of the residual avoidance is smaller than observed in the wild type, but comparable with that in the *phot1* mutant. Chimeric proteins containing an N-terminus of phot1 and a C-terminus of phot2, or vice versa, are both capable of triggering chloroplast avoidance (Aihara *et al.*, 2008). However, the protein bearing the phot1 N-terminus shows increased light sensitivity. The authors propose that the avoidance response is suppressed for phot1 by a mechanism requiring both the N- and C-terminal parts of the protein. This active suppression mechanism agrees with the observed transient character of the avoidance reaction occurring even upon continuous light illumination. This suppression probably requires the recruitment of some additional factors, which is reflected in the time lag between the onset of the signal and its quenching, thereby allowing the transient reaction to take place.

### The role of PP2A in chloroplast movements

Two different modes of action have been assigned to PP2A in relation to phototropin signaling. First, it dephosphorylates phot2 via a direct interaction between phot2 and the PP2A scaffolding subunit A1 (RCN1). As a consequence, the *rcn1-1* mutation enhances phot2 phosphorylation and phototropin-mediated responses in seedlings (Tseng and Briggs, 2010). Later, on the basis of impaired chloroplast avoidance in the mutant of the catalytic subunit *pp2a-2*, PP2A was proposed to be involved in downstream events in the movement mechanism (Wen *et al.*, 2012). However, in our experimental system, the *pp2a-2* mutant does not differ from the wild type in terms of movement responses, even though the same SALK line as described by Wen *et al.* (2012) was used. Given the impact of phosphatase inhibitors on chloroplast movements (Wen *et al.*, 2012; our unpublished data), it appears that phototropin-regulated dephosphorylation events are important for the movement mechanism, but phosphatases responsible for this process remain to be determined. None of the B' subunits examined here specifically and exclusively participates in the regulation of chloroplast relocations, despite their involvement in other high light acclimation responses (Konert *et al.*, 2015). On the other hand, the lack of phenotypes in the mutants may result from some redundancy of PP2A subunits.

The *rcn1* mutant shows a decreased amplitude of the accumulation phase in biphasic responses to longer pulses (Fig. 5), which can be interpreted as a shift towards a longer pulse response. This effect may be a consequence of increased expression of both phototropins at the protein level (Fig. 6) observed in the *rcn1* mutant. In the experimental system herein, the *rcn1* mutant showed slightly delayed dephosphorylation of phot2 as compared with the wild type. Nevertheless, the phosphorylation of both phototropins decreases in darkness even in *rcn1*, implying that some other phosphatases or PP2A subunits are involved in the dephosphorylation of these photoreceptors.

It should be pointed out that dephosphorylation studies reported here were conducted in a light regime different from the one used for eliciting chloroplast movements. Phototropin phosphorylation was induced by 1h of blue light at 120 µmol m^−2^ s^−1^, whereas movements were elicited by pulses of the same light intensity lasting only up to 20s. This longer irradiation time was chosen to saturate phototropin phosphorylation in leaves in order to facilitate the observation of any potential changes in the dephosphorylation kinetics. Phosphorylation of phot1 has been reported to occur after short irradiation with relatively low blue light: 1min of 55 µmol m^−2^ s^−1^ in microsomal fractions isolated from seedlings (Liscum and Briggs, 1995) or as low as 5 µmol m^−2^ s^−1^ for 30s in etiolated seedlings (Inoue *et al.*, 2008). Phosphorylation of phot2 was demonstrated after irradiation with blue light at 500 µmol m^−2^ s^−1^ for 1min (Inoue *et al.*, 2011). Therefore, brief pulses of blue light used here to elicit chloroplast movement should trigger autophosphorylation of at least a fraction of phototropins. Factors that affect phototropin dephosphorylation ought to play a role irrespective of the saturation level of phototropin phosphorylation.

The relationship between phototropin phosphorylation and signaling seems to be complex. Mutants with altered phosphorylation sites in the activation loop still display a typical mobility shift (Inoue *et al.*, 2011), despite the fact that these phototropin molecules are unable to trigger signaling. Phosphomimic mutants require light for their activity (Inoue *et al.*, 2011). This suggests that phosphorylation alone is not sufficient for signal transduction, and that light-driven structural changes are also necessary. Thus, the maintenance of phosphorylation would not be sufficient to sustain signaling, unless it is accompanied by a stabilization of the light-induced conformational changes in the phosphorylated molecule. However, the impact of photoreceptor phosphorylation on its molecular dynamics has not yet been established.

### Conclusion

Chloroplast responses to light pulses are an excellent tool for examining molecular aspects of photoreceptor activation during signal transduction. The analysis of phototropin mutants reveals alterations in chloroplast reactions to pulses. The most prominent effect is observed in the *phot2* mutant, where chloroplast accumulation is enhanced. The formation of both homo and heterodimers by phototropins supports the hypothesis of photoreceptor co-operation in eliciting chloroplast responses to light. Thus, mutant phenotypes appear to be the consequence of a loss of interaction between phototropins rather than antagonism between them.

## Supplementary data

Supplementary data are available at *JXB* online

Table S1. Sequence of primers used for genotyping.

Table S2. Primers used for Gateway cloning.

Table S3. Plasmids used for preparation of BiFC and MYTH constructs

Figure S1 Version of Fig. 4 . containing the error bars.

Figure S2. Characterization of the *pp2a-2* (SALK_150673) line.

Figure S3. Negative controls for BiFC.

Figure S4. Expression of proteins in the BiFC experiment.

Figure S5. MYTH assays using full-length phototropins as baits and truncated phototropins as preys.

Supplementary Data
