# Corrigendum: Fine tuning chloroplast movements through physical interactions between phototropins

**DOI:** 10.1093/jxb/ery008

**Published:** 2018-02-06

**Authors:** Olga Sztatelman, Justyna Łabuz, Paweł Hermanowicz, Agnieszka Katarzyna Banaś, Aneta Bażant, Piotr Zgłobicki, Chhavi Aggarwal, Marcin Nadzieja, Weronika Krzeszowiec, Wojciech Strzałka, Halina Gabryś

**Affiliations:** 1 Department of Plant Biotechnology, Faculty of Biochemistry, Biophysics and Biotechnology, Jagiellonian University, Gronostajowa, Krakow, Pol; 2 Institute of Biochemistry and Biophysics, Polish Academy of Sciences, Pawińskiego, Warsaw, Pol; 3 Malopolska Centre of Biotechnology, Jagiellonian University, Gronostajowa, Krakow, Pol

*Journal of Experimental Botany,* Vol. 67, No. 17 pp. 4963–4978, 2016 doi: 10.1093/jxb/erw265

The original version of this paper contained unspecific funding information. In order to properly accredit funders, the information has been updated as follows:

In the ‘Acknowledgements’ section, the sentence “The work was funded within the EU framework of FP7, Marie Curie ITN CALIPSO [GA 2013-ITN-607-607].” has been changed to read “The work was funded by financial resources for science in the years 2013–2017, allocated to the realization of a co-financed international project Marie Curie ITN CALIPSO [GA 2013-ITN-607-607] within the EU framework of FP7.”

The authors would like to apologise for this error.

