# Supplementary material for: Fine tuning chloroplast movements through physical interactions between phototropins
Source: J Exp Bot. 2016 Jul 12;67(17):4963–78. doi: 10.1093/jxb/erw265 (PMC5014152; doi:10.1093/jxb/erw265)
Supplement: Supplementary Data [file supp_67_17_4963__index.html]

Fine tuning chloroplast movements through physical interactions between phototropins — Fine tuning chloroplast movements through physical interactions between phototropins — Supplementary Data 

# Fine tuning chloroplast movements through physical interactions between phototropins

## Supplementary Data

Data files

- supplementary\_tables\_S1\_S3\_Figures\_S1\_S5.pdf - Supplementary Data
