# Supplementary material for: Fine tuning chloroplast movements through physical interactions between phototropins
Source: J Exp Bot. 2016 Jul 12;67(17):4963–78. doi: 10.1093/jxb/erw265 (PMC5014152; doi:10.1093/jxb/erw265)
Supplement: Supplementary Data [file supp_erw265_supplementary_tables_S1_S3_Figures_S1_S5.pdf]

Supplementary Material

The interplay of phototropins in signaling to chloroplast movements.

Olga Sztatelman, Justyna Łabuz, Paweł Hermanowicz, Agnieszka Katarzyna Banaś, Aneta Bażant, Piotr Zgłobicki, Chhavi Aggarwal, Marcin Nadzieja, Weronika Krzeszowiec, Wojciech Strzałka, Halina Gabryś

Table S1. Sequence of primers used for genotyping.

Primer name	Nucleotide sequence (5' - 3')
rcn1RP	AAACATAGCCACACGCATTTC
rcn1LP	GGCCAGCCAGTTAGGTATAGG
pp2A-2RP	TCCAAGAATTCACCATTTTGG
pp2A-2LP	GAGGTCCCGAGTTCAATTCTC
Lba1	TGGTTCACGTAGTGGGCCATCG

Table S2. Primers used for Gateway cloning

Primer name	Nucleotide sequence (5' - 3')
attb1_phot1_for	GGGGACAAGTTTGTACAAAAAAGCAGGCTTCATGGAACCAACAGAAAAACC
attb2_phot1_rev	GGGGACCACTTTGTACAAGAAAGCTGGGTCAAAAAAACATTTGTTTGCAG
attb2_phot1_rev_nostop	GGGGACCACTTTGTACAAGAAAGCTGGGTCAAAAACATTTGTTTGCAGATC
attb1_phot2_f	GGGGACAAGTTTGTACAAAAAAGCAGGCTTCATGGAGAGAGGCCAAGAGCC
attb2_phot2_r	GGGGACCACTTTGTACAAGAAAGCTGGGTCTTAGAAGAGGTCAATGTCCAAG
attb2_phot2_rev_nostop	GGGGACCACTTTGTACAAGAAAGCTGGGTCGAAGAGGTCAATGTCCAAGTC
attB1_phot1_Cterm_for	GGGGACAAGTTTGTACAAAAAAGCAGGCTTCATGACACCAGAGGATTTATGGGC
attB2_phot1_Nterm_rev	GGGGACCACTTTGTACAAGAAAGCTGGGTCGTTGGCATCAGGAAGTTCTCGAAC
attB1_phot2_Cterm_for	GGGGACAAGTTTGTACAAAAAAGCAGGCTCGCATCATTTCAAACCAATAAAACC
attB2_phot2_Nterm_rev	GGGGACCACTTTGTACAAGAAAGCTGGGTCTAGTCCCACTGTTTCTCCACTC

Table S3. Plasmids used for preparation of BiFC and MYTH

Plasmid name	Short description	Reference
pDONR221	Gateway entry vector	
pH7m34GW	MultiSite Gateway intermediary vector (three fragment recombination plus terminator) backbone vector for C-terminal fusion, used to obtain 35S-PHOT1(2)-nGFP	(Karimi *et al.*, 2005)
pK7m34GW	MultiSite Gateway intermediary vector (three fragment recombination plus terminator) backbone vector for C-terminal fusion used to obtain 35S-PHOT1(2)-cGFP	(Karimi *et al.*, 2005)
pH7m24GW2	MultiSite Gateway intermediary vector (two fragment recombination plus terminator) backbone vector for N-terminal fusion	(Karimi *et al.*, 2007)
pK7m24GW2	MultiSite Gateway intermediary vector (two fragment recombination plus terminator) backbone vector for N-terminal fusion used to obtain 35S- cGFP-PHOT1(2)	(Karimi *et al.*, 2007)
pEN-L4-2-R1	Gateway entry vector with 35S promoter used for multisite gateway reaction	(Karimi *et al.*, 2007)
pEN-R2-teGFP-L3	Gateway entry vector with cGFP used for multisite gateway reaction to obtain cGFP fusion at the C-terminus of the protein	(Karimi *et al.*, 2007)
pEN-R2-heGFP-L3	Gateway entry vector with nGFP used for multisite gateway reaction to obtain nGFP fusion at the C-terminus of the protein	(Karimi *et al.*, 2007)
pEN-L4-heGFP-R1	Gateway entry vector with nGFP used for multisite gateway reaction to obtain nGFP fusion at the N-terminus of the protein	(Karimi *et al.*, 2007)
pEN-L4-teGFP-R1	Gateway entry vector with cGFP used for multisite gateway reaction to obtain cGFP fusion at the N-terminus of the protein	(Karimi *et al.*, 2007)
pPR3_Gateway	Gateway prey vector used for MYTH	(Strzalka *et al.*, 2015)
pDHB1_Gateway	Gateway bait vector used for MYTH	(Strzalka *et al.*, 2015)

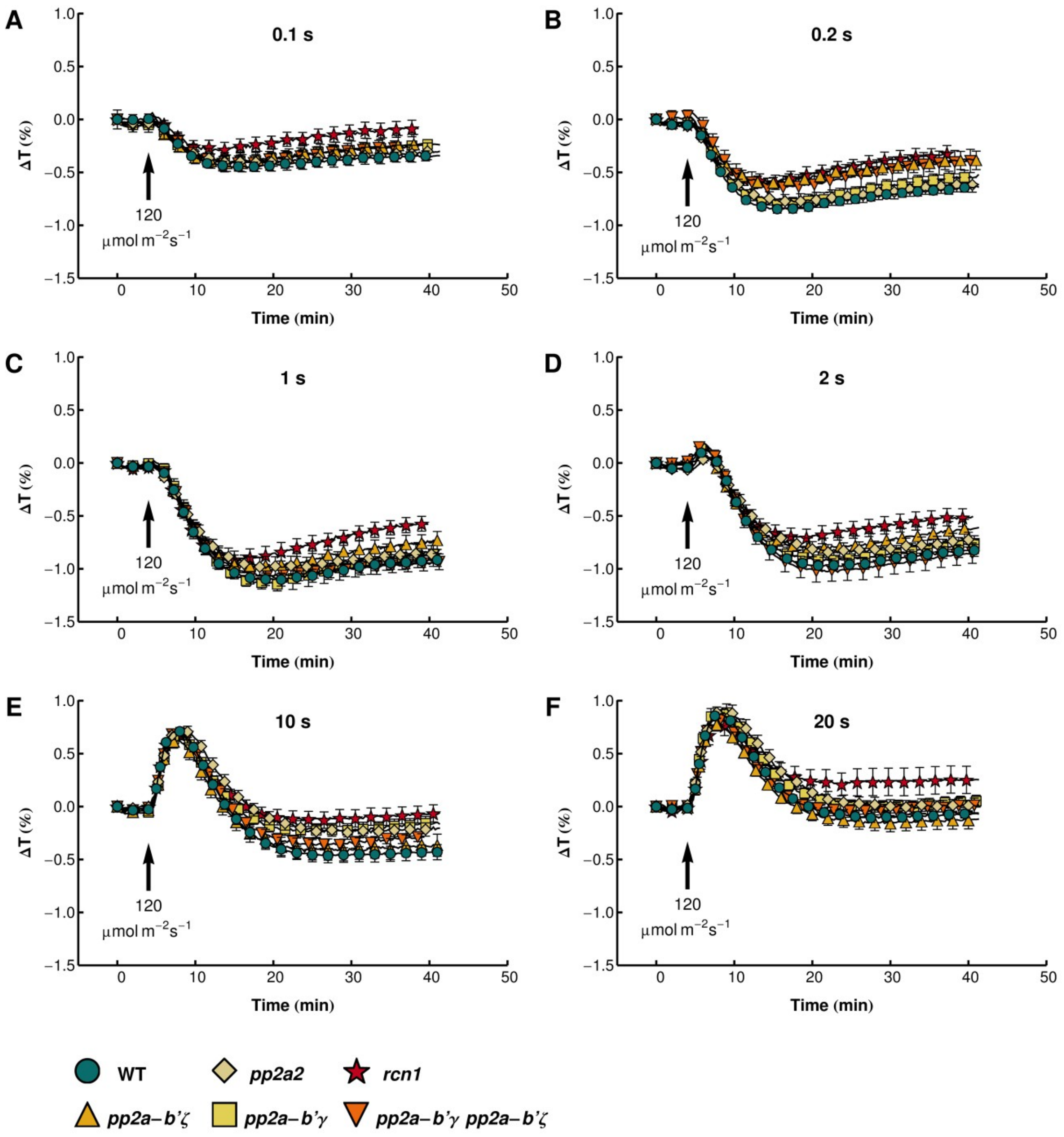

Figure S1 Figure 4 with error bars. Chloroplast movements in response to strong blue light pulses in wild type *Arabidopsis* and mutants in selected subunits of PP2A phosphatase. Time course of changes in red light transmittance were recorded before and after a blue light pulse of 120 µmol·m^{-2}·s^{-1}and duration specified in the figure. Each data point is an average of at least 7 measurements. Error bars- SE.

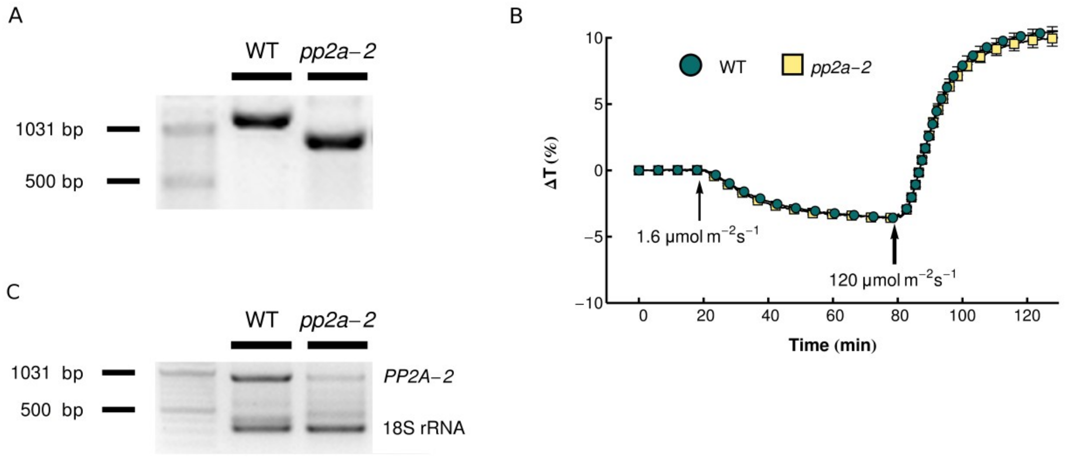

Figure S2 A- Genotyping of the *pp2a-2* (SALK_150673) line. Predicted product size for the wild type: 1134 bp, product size for the mutant: 589-889 bp (calculated by T-DNA Primer Design tool: http://signal.salk.edu/tdnaprimers.2.html). DNA was separated in 1% agarose in TAE buffer and stained with Midori Green. B- Time-course of chloroplast movements in response to continuous weak blue light (1.6 µmol·m^{-2}·s^{-1}) followed by strong blue light (120 µmol·m^{-2}·s^{-1}) in wild type *Arabidopsis* and *pp2a-2* (SALK_150673) mutant. The dark transmittance level was recorded for 20 minutes. The onset of each light intensity is marked by arrows. Each data point is an average of at least 7 measurements. Error bars- SE. C- The expression of *PP2A-2* in WT and homozygous *pp2a-2* (SALK_150673) leaves. 18S RNA served as an internal standard.

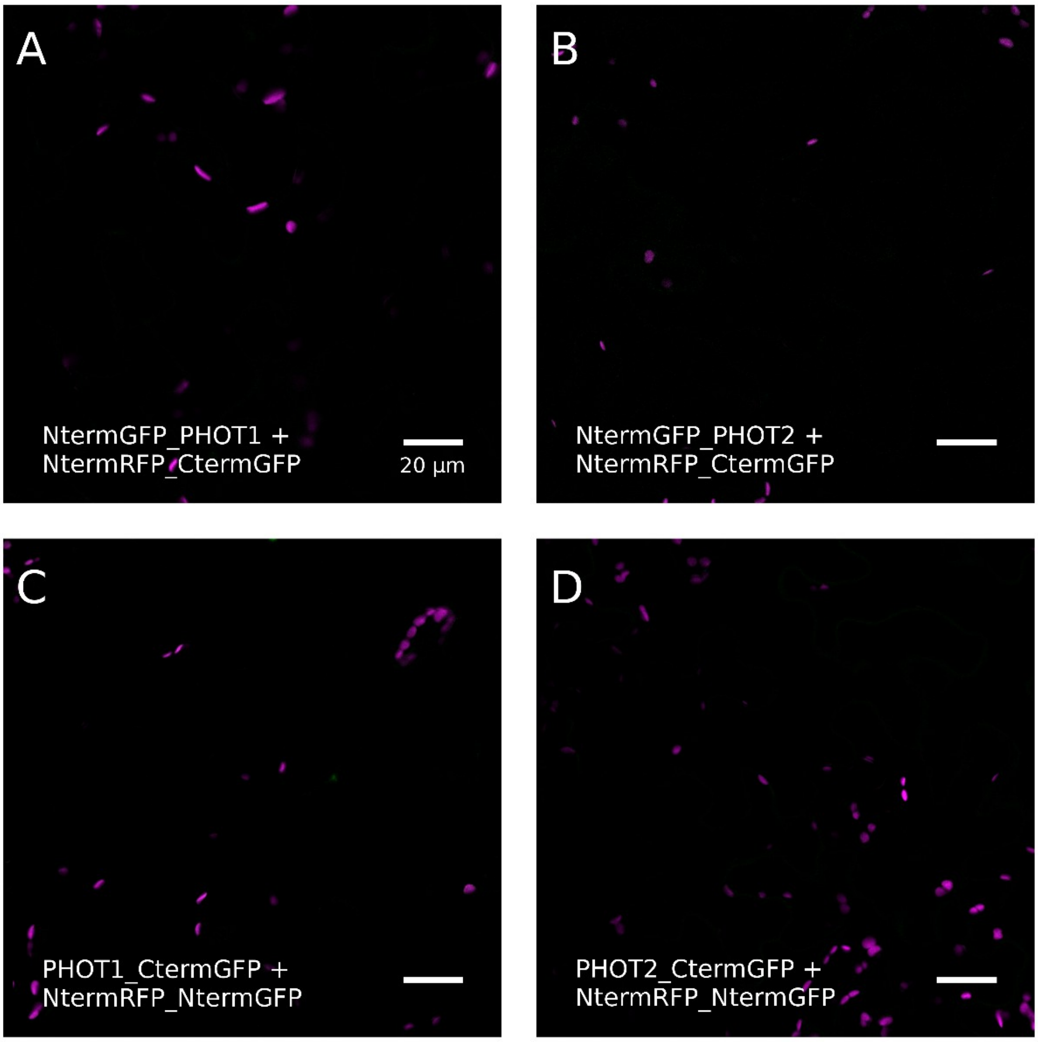

Figure S3 Confocal images of *N. benthamiana* epidermal cells transiently co-expressing a GFP fragment fused with PHOTs and the second GFP fragment fused with 150 initial amino-acids of RFP (Nterm-RFP) as a control protein not interacting with phototropins. Pairs used as negative control are in the following configurations: A- NtermGFP_PHOT1 and NtermRFP_CtermGFP, B- NtermGFP_PHOT2 and NtermRFP_CtermGFP, C- PHOT1_CtermGFP and NtermRFP_NtermGFP and D- PHOT2_CtermGFP and NtermRFP_NtermGFP. Chlorophyll autofluorescence in magenta, reconstituted GFP fluorescence in green. Scale bar- 20 µm. The results represent one of three independent biological replicates.

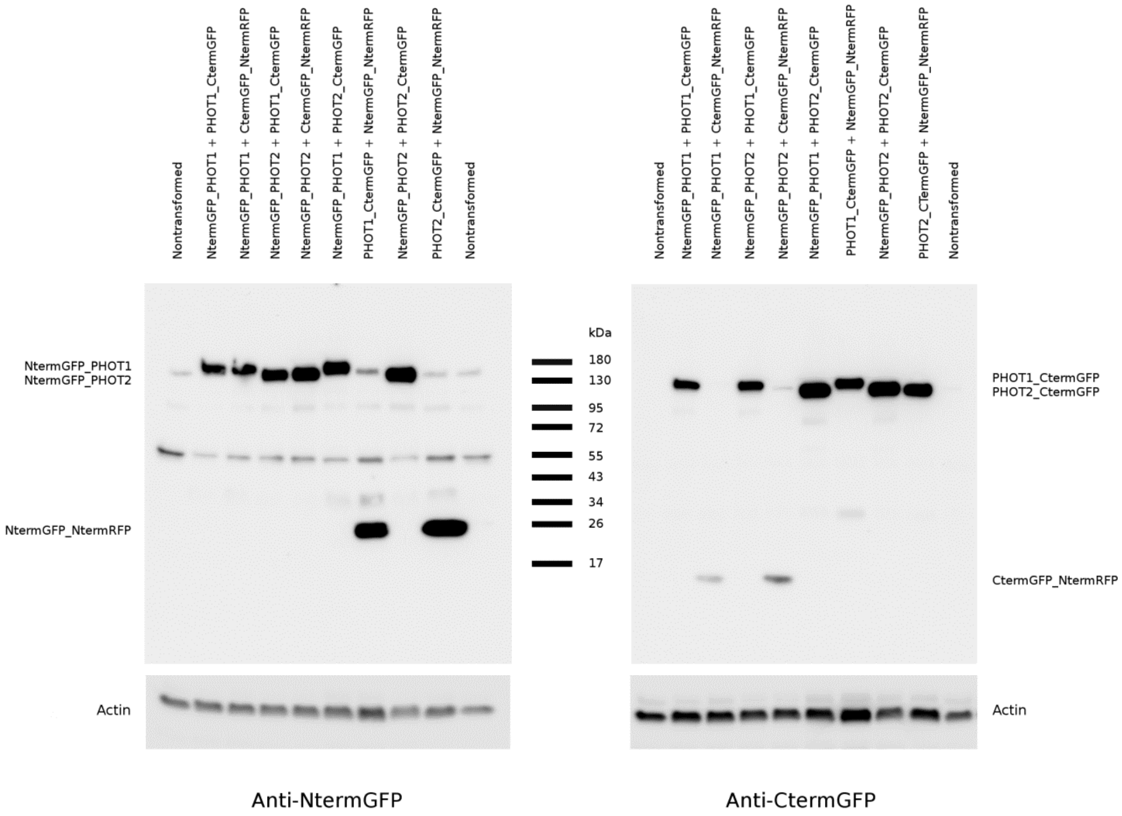

Figure S4 Expression of phototropins fused with N- or C- terminal GFP parts and control constructs in transiently transformed *N. benthamiana* epidermal cells. Left: blots probed with the antibody against the N-terminal part of GFP (Living Colors, Clonetech). Right: blots probed with the antibody against the C-terminal part of GFP (Santa Cruz Biotechnology). Below: loading control: blots probed with antibody against actin.

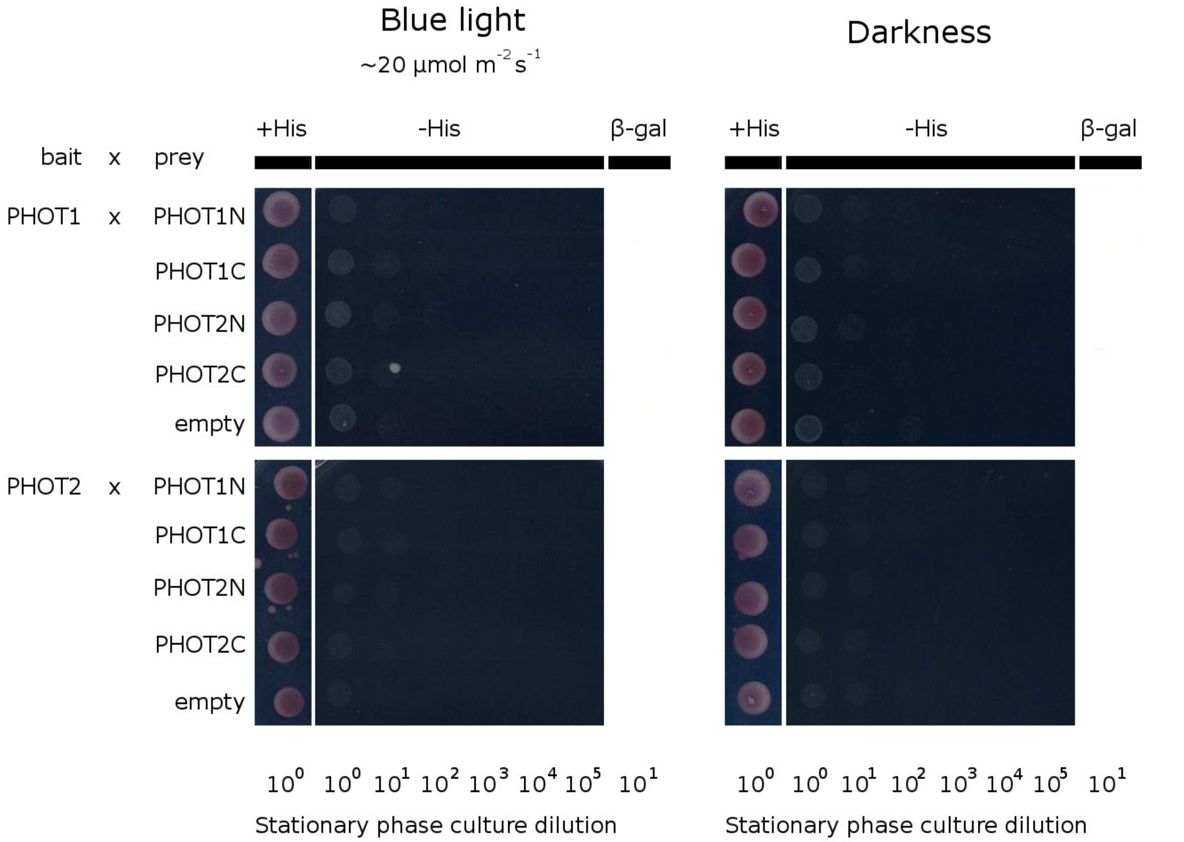

Figure S5 Phototropin interactions tested with MYTH. Full length phototropins were used as baits and their N/C-terminal parts were used as preys. Overnight cultures of transformed yeasts were plated on the solid SC-Leu-Trp (+His) medium serving as a control, SC-Leu-Trp-His (-His) solid selection medium supplemented with 5 mM 3-aminotriazol (3-AT) or YPAD solid medium to perform β-galactosidase filter lift-off assay. In each case the yeast plated on solid media were cultured either in darkness or under blue light (~20 µmol·m^{-2}·s^{-1}, 470 nm) in 30°C for 3 days. For all bait/prey constructs a co-transformation with empty prey/bait vectors was performed to avoid false-positive signals being a result of a non-specific self-activation. The results represent one of at least three independent biological replicates.

References

Karimi M, Bleys A, Vanderhaeghen R, Hilson P. 2007. Building blocks for plant gene assembly. Plant Physiology **145**, 1183–91.

Karimi M, De Meyer B, Hilson P. 2005. Modular cloning in plant cells. Trends in Plant Science **10**, 103–5.

Strzalka WK, Aggarwal C, Krzeszowiec W, Jakubowska A, Sztatelman O, Banas AK. 2015. Arabidopsis PCNAs form complexes with selected D-type cyclins. Frontiers in Plant Science **6**, 1–11.
